# Interplay of Val66Met and BDNF methylation: effect on reward learning and cognitive performance in major depression

**DOI:** 10.1186/s13148-021-01136-z

**Published:** 2021-07-29

**Authors:** J. Bakusic, E. Vrieze, M. Ghosh, D. A. Pizzagalli, B. Bekaert, S. Claes, L. Godderis

**Affiliations:** 1 Centre for Environment and Health, Department of Public Health and Primary Care, KU Leuven (University of Leuven), Kapucijnenvoer 35, 3000 Leuven, Belgium; 2 Psychiatry Research Group, Department of Neuroscience, KU Leuven, Leuven, Belgium; 3 Department of Psychiatry, Harvard Medical School, Boston, MA USA; 4 Department of Forensic Medicine, Laboratory of Forensic Genetics and Molecular Archaeology, KU Leuven, Leuven, Belgium; 5 Department of Imaging & Pathology, KU Leuven, Leuven, Belgium; 6 IDEWE, External Service for Prevention and Protection at Work, Heverlee, Belgium

**Keywords:** Depression, Epigenetics, DNA methylation, BDNF, Reward learning, Cognitive performance

## Abstract

**Background:**

There is a growing interest in the role of brain-derived neurotrophic factor (BDNF) in major depressive disorder (MDD). BDNF potentially exhibits opposite effects in the pathways linked to anhedonia and reward learning on the one hand and cognitive performance, on the other hand. However, the epigenetic mechanisms behind this remain unknown. In the present study, we aimed to investigate the interplay of DNA methylation of different *BDNF* exons and the common Val66Met polymorphism on anhedonia, reward learning and cognitive performance in MDD.

**Methods:**

We recruited 80 depressed patients and 58 age- and gender-matched healthy controls. Participants underwent clinical assessment including neuropsychological testing and a probabilistic reward task to assess reward learning. Val66Met polymorphism and DNA methylation of *BDNF* promoters I, IV and exon IX were assessed from whole blood derived DNA, using pyrosequencing.

**Results:**

*BDNF* promoter I methylation was lower in MDD patients (*p* = 0.042) and was negatively associated with self-reported anhedonia. In depressed patients, both Val66Met polymorphism and DNA methylation of promoter I were significantly associated with reward bias (*p* < 0.050 and *p* = 0.040, respectively), without an interaction effect. On the other hand, methylation of exon IX had a negative impact on executive functioning (*p* = 0.002) and mediated the effect of Val66Met on this outcome in patients with MDD.

**Conclusions:**

Our results provide the first evidence of Val66Met susceptibility to differential epigenetic regulation of *BDNF* exons in reward learning and executive functioning in MDD, which needs to be further explored.

**Supplementary Information:**

The online version contains supplementary material available at 10.1186/s13148-021-01136-z.

## Background

Major depressive disorder (MDD) is one of the leading causes of disability worldwide, affecting more than 150 million people [1]. In addition, the efficacy of the current treatment for MDD is suboptimal, and only about 30% of patients will achieve adequate remission after optimal treatment according to consensus guidelines [2]. Efforts to improve treatment strategies are, among others, hindered by the insufficient knowledge about the underlying pathophysiological mechanisms [3].

Over the past years, molecular studies of depression have moved beyond the monoamine hypothesis. Novel observations led to the “neurotrophic hypothesis of depression”, highlighting the functional significance of alterations in neurotrophic factors, particularly the brain-derived neurotrophic factor (BDNF) [4]. BDNF regulates neural development in early stages of life and plays a critical role in neural differentiation, and neurite and synaptic growth in adult brain [5]. In addition, by regulating synaptic plasticity and neurogenesis in different brain regions, BDNF seems to have a pivotal role in memory acquisition and consolidation [6].

Findings from multiple individual studies as well as meta-analyses suggest that depressed patients are characterized by decreased peripheral concentrations of BDNF compared to healthy controls [7–10]. Moreover, the functional significance of reduced BDNF levels in MDD was demonstrated in studies showing that this blunting was associated with neural atrophy and loss in key limbic regions such as hippocampus and prefrontal cortex [4, 11]. These alterations are in line with the observed reduced performance of depressed patients in cognitive tasks [11]. In contrast, increased BDNF levels in brain regions involved in mesolimbic dopaminergic circuit, such as the ventral tegmental area (VTA) and nucleus accumbens (NA), were shown to induce stress-related depressive-like behaviour in animal studies [12, 13]. Mesolimbic BDNF signalling seems to play a role in a range of motivation and reward-related behaviours [14] and has therefore been implicated in the pathophysiology of MDD, particularly with regard to loss of pleasure or anhedonia [15].

A growing body of research emphasizes the significance of the interplay between genetic vulnerability, reflected in the existence of single-nucleotide polymorphisms (SNPs), and epigenetic regulation of the *BDNF* gene in psychopathology. The *BDNF* gene has a complex structure, containing 11 exons and 9 have functional promoters that are brain region- and tissue-specific [16]. In particular, a large number of studies focused on investigating the rs6265 SNP as a potential risk biomarker in MDD [17]. This SNP is located at exon IX and causes guanine (G) to adenosine (A) replacement at codon 66, resulting in substitution of valine (Val) to methionine (Met) and a consequent impairment of the activity-dependent release of BDNF [18]. Although there is some evidence of the role of rs6265 in depression, the findings are inconsistent, especially in terms of identifying which of the two allele (Val or Met) indicates higher vulnerability [17].

One of the possible reasons for this inconsistency is the potential moderating effect of epigenetic regulation on the effect of the polymorphism on clinical outcomes. Epigenetic regulation of *BDNF* in depression has been increasingly studied, particularly DNA methylation [19]. Briefly, DNA methylation involves transfer of a methyl group to the CpG nucleotides, which mainly leads to gene silencing when located in the gene promoter region [20]. In the context of *BDNF*, DNA methylation of promoters of exon I and IV has been the most extensively studied [21, 22]. Increased *BDNF* methylation in depressed patients compared to healthy subjects has been reported in several studies, mainly in promoter of exon I and IV [23–26]. Most of these studies assessed purely differences in *BDNF* methylation between depressed patients and healthy controls. In addition, the interplay between Val66Met and *BDNF* methylation has been somewhat studied in the general population, showing that Val66Met affects DNA methylation of different individual CpGs in the *BDNF* gene [27, 28].

In contrast, the moderating and mediating roles of *BDNF* methylation on the association of Val66Met and specific functional outcomes corresponding to differential BDNF pathways in MDD remain largely unexplored. Despite the well-established role of BDNF in anhedonia and reward circuits on the one hand and cognitive performance on the other hand, the effect of interplay between the rs6265 SNP and *BDNF* methylation on these outcomes in MDD remains poorly understood. To the best of our knowledge, there are no studies investigating genetic and epigenetic regulation of *BDNF* in the context of anhedonia and reward learning in MDD. Regarding cognitive performance, only one study focused on the effect of rs6265 and methylation of *BDNF* exon I and IV [29], but without assessing methylation of exon IX, which contains the polymorphism. Previously, we found methylation of exon IX to be highly correlated with rs6265 and serum BDNF concentration [30] and changes in methylation of this exon were previously associated with changes in BDNF expression in MDD patients [31]. Therefore, in the present study, we aimed to investigate the genetic (rs6265) and epigenetic regulation of *BDNF* exon I, IV and IX in MDD and their impact on two major outcomes: (1) anhedonia and reward learning and (2) cognitive performance.

## Methods

### Study population

Eighty depressed patients and 58 healthy control subjects, matched for age and gender, were included. All patients were hospitalized at the University Psychiatric Centre of the University of Leuven in Belgium. A detailed recruitment procedure and inclusion/exclusion criteria were described previously [32, 33]. Briefly, the MDD diagnosis and psychiatric comorbidity were evaluated by a psychiatrist using the Structured Clinical Interview for DSM-IV-TR (SCID-I) [34]. Patients with other mood spectrum disorders, substance abuse or unstable medical conditions were excluded. Almost all patients started antidepressant treatment prior to admission to the hospital. During follow-up, treatment was not standardized and patients were treated with psychopharmacology and/or psychotherapy. Healthy participants did not meet criteria for any current or past psychiatric disorder or unstable medical conditions and were assessed only at baseline.

The study was approved by the UZ Leuven Medical Ethics Committee and all participants signed an informed consent.

## Clinical and neuropsychological assessment

During the psychiatric interview, the 17-item Hamilton Rating Scale for Depression (HDRS) [35] was used to assess the severity of depression and the semi-structured trauma questionnaire (STI) was used to assess early life stress (ELS) [36]. Anhedonia was assessed using self-reported Snaith–Hamilton Pleasure Scale (SHAPS) [37] and positive and negative affect were measured using the Positive and Negative Affect Schedule (PANAS) [38].

To assess different aspects of cognitive performance, a battery of neuropsychological tests was administered. This included: (1) executive functioning/cognitive interference: Stroop Color and Word Test (SCWT) [39], (2) processing speed: Trial Making Test A (TMT-A) [40], (3) attention: Digit Span Forward (DS-F) [41] and (4) executive control: Trial Making Test B (TMT-B) [40] and Digit Span Backward (DS-B) [41]. All scores were transformed to percentiles, based on age, gender and education level, according to the appropriate reference categories [40, 42].

## Reward task

A computerized reward learning task was used to measure participants’ ability to modulate behaviour as a function of reward [43]. The task has been previously described in detail [33, 44] and has been shown to objectively measure reward responsiveness in healthy individuals and depressed patients [43]. Briefly, the task lasted approximately 25 min and included 300 trials, divided in 3 blocks of 100 trials. In each trial, a cartoon face with either short or long mouth appeared for 100 ms. Participants were instructed to indicate which type of mouth was presented, and a monetary award was given for approximately 40 correct answers. An asymmetric reinforcement ratio was used to induce a response bias, meaning that subjects received a reward 3 times more frequently for correct identification of one mouth (the “rich stimulus”) than for correct identification of the other mouth (the “lean stimulus”). Before the task, participants were instructed that the goal of the task is to win as much money as possible. Response bias (RB) towards the rich stimulus was used as a measure of reward learning and was calculated as the difference in RB across Blocks (1, 2 and 3).

## Genetic and epigenetic analysis

DNA was extracted from whole blood and the quantity and purity of DNA were determined using a NanoDrop spectrophotometer. Next, DNA samples were bisulphite-converted using the EZ-96 DNA Methylation-Gold™ Kit (#D5008, Zymo Research), according to the manufacturer’s protocol. Polymerase chain reaction (PCR) was used to amplify the bisulphite-converted DNA sequences selected for genetic and epigenetic analyses. For this, 1 μL of converted DNA was amplified by PCR in a total volume of 25 μL containing 0.2 μM of primers and 2 × Qiagen PyroMark PCR Master Mix (#978703, Qiagen). Pyrosequencing was performed using Pyro Gold reagents (#970802, Qiagen) on the PyroMark Q24 instrument (Qiagen) following the manufacturer’s instructions. Pyrosequencing results were analysed using the PyroMark analysis 2.0.7 software (Qiagen). The analysed sequences include 8 CpGs in the promoter of exon I (4 in promoter Ia and 4 in promoter Ib), 7 CpGs in promoter of exon IV and 5 CpGs in a coding region in exon IX (including Val66Met polymorphism) of the *BDNF* gene. We focused on DNA methylation of these specific regions due to their relevance for *BDNF* expression as well as the fact that these transcripts are highly abundant in both blood (leucocytes) and the brain tissue (hippocampus) [45]. Primer validation was performed through gel electrophoresis using MultiNA (Shimadzu Benelux BV, Belgium). Control DNA was used to perform gel electrophoresis and to validate each pyrosequencing analysis. All samples were randomized prior to DNA methylation analysis.

A detailed protocol with all analysed amplicons, PCR and sequencing primers is provided in the Additional file 1 and has been previously described in detail [46].

## Statistical analysis

Comparisons of socio-demographic and clinical variables between the control and the depression group were performed using an independent sample *t*-test for continuous variables and Chi-Square test for categorical variables. Genotype differences between the groups were tested using Chi-Square test. The effect of polymorphism on DNA methylation as well as DNA methylation differences between groups was tested using Mann–Whitney *U* test due to data skewness. Methylation was analysed as average methylation percent across different CpGs within each assay to avoid multiple testings. CpG-specific analyses were only performed in the between-group comparison and to test the effect of polymorphism on DNA methylation of specific CpGs. As these analyses were explorative in nature, no additional correction for multiple testing was applied.

Explorative associations between DNA methylation and clinical variables (anhedonia, depression severity and cognitive performance) were tested using Spearman’s correlation. The observed correlations were further tested in univariate linear models, with appropriate covariates. The effect of the polymorphism on clinical variables (anhedonia, depression severity, cognitive performance) was tested either using an independent sample *t*-test or Mann–Whitney *U* test, depending on the distribution of the outcome variable. In case we observed an association between both the polymorphism and DNA methylation and any of the cognitive performance outcomes, we tested potential mediation effect of DNA methylation on this association, by using the PROCESS macro of SPSS version 21.0, developed by Hayes [10]. The mediation effect was interpreted using the bias-corrected bootstrap confidence intervals, whereby the mediation effect was considered significant when the bias-corrected 95% confidence intervals did not contain zero.

To test the association between polymorphism/DNA methylation and response bias, we applied a repeated measures ANOVA with response bias as the outcome variable and with Block (1, 2, 3) and polymorphism/DNA methylation as predictors. This model was also used to test for potential interactions between polymorphism and DNA methylation on response bias. The Greenhouse–Geisser correction was used when the sphericity assumption was violated.

All statistical analyses were performed using SPSS software package, version 26.0. All tests were two-sided, and the significance level was set at 0.05.

## Results

### Population characteristics

Socio-demographic and clinical characteristics of all participants are presented in Table 1. Socio-demographic features were comparable between the groups, except from education level, which differed significantly (*X*^2^ = 19.3, *p* < 0.001). As expected, depressed patients had higher levels of anhedonia and more frequently reported early life stress events (*ps* < 0.001). Regarding the cognitive performance tests, relative to the healthy controls, depressed patients had significantly poorer performance in SCWT, TMT-A and TMT-B (all *ps* < 0.001) but not in DS-F (*p* = 0.098) nor DS-B (*p* = 0.411).

**Table 1:** Socio-demographic and clinical characteristics of participants at baseline

	Control group (*N* = 58)	Depressed group (baseline, *N* = 80)	Significance
Age (year, mean ± SD)	45.5 ± 12.0	45.4 ± 12.1	*t* = 0.08
			*p* = 0.93
Gender (*N*, female/male)	31/27	47/33	X^2^ = 0.38
			*p* = 0.53
Educational level (low/high)^a^	13/45	48/32	X^2^ = 19.3
			*p* < 0.001**
*Antidepressant use (%)*			
None	96.6	4	-
SSRI	1.7	41	
SNRI	0	34	
Other (TCA, mirtazapine, bupropion)	1.7	21	
Early life stress event (% yes)^b^	10.3	38.0	X^2^ = 13.18, *p* < 0.001**
Depression severity (HDRS)	0.6 ± 1.3	16.9 ± 5.0	*t* = 27.54, *p* < 0.001**
Anhedonia (SHAPS)	19.2 ± 4.1	35.7 ± 7.6	*t* = 16.58, *p* < 0.001**
*Cognitive performance (percentile* ± *SD)*			
SCWT	65.3 ± 25.3	29.6 ± 2.5	*t* = 8.28, *p* < 0.001**
TMT-A	67.8 ± 22.6	35.2 ± 23.0	*t* = 8.26, *p* < 0.001**
TMT-B	65.2 ± 26.6	38.3 ± 26.4	*t* = 5.86, *p* < 0.001**
DS-F	34.3 ± 25.5	29.7 ± 27.8	*t* = 0.99, *p* = 0.32
DS-B	45.9 ± 25.9	42.0 ± 25.5	*t* = 0.87, *p* = 0.39

*P*-values are derived from statistical analysis using independent sample *t*-test for continuous variables or Chi-Square test for categorical variables

^a^Low education = finished secondary school or less; High education = any additional education after secondary school

^b^Early life stress event: assessed by Structured Trauma Inventory Scale (STI)

HDRS: Hamilton Rating Scale for Depression; SHAPS: Snaith–Hamilton Pleasure Scale; SCWT: Stroop Color and Word Test; TMT-A: Trial Making Test part A; TMT-B: Trial Making Test part B; DS-F: Digit Span Forward; DS-B: Digit Span Backward

## Val66Met Polymorphism and depression

Evaluating the polymorphism data, depressed patients and healthy subjects did not differ significantly in the number of Met carriers (36.2% in the MDD group compared to 32.8% in the control group, *X*^*2*^ = 0.181, *p* = 0.720). In addition, Val66Met polymorphism was not associated with any clinical features (depression severity, anhedonia), neither in the whole sample nor in the depression group separately (all *ps* > 0.05, data not shown). I n terms of the effect of Val66Met on *BDNF* methylation in the whole sample (both groups), the presence of a Met allele had a negative effect on the average methylation of the exon IX (mean difference 8.78%, *p* < 0.001), as well as 4 out of 5 individual CpGs in this region (Additional file 1: Table S2). In addition, Met carriers had lower average methylation of exon Ia (mean difference 0.35%, *p* = 0.006) and exon Ib (mean difference 0.28%, *p* = 0.014) as well as several individual CpGs in these two regions (presented in Additional file 1: Table S2) in the overall sample. For exon IV, only association with one CpG was found (CpG5: mean difference 0.34%, *p* = 0.011). Group (depression *vs.* control) was not a moderator of any of these associations (all *ps* > 0.05).

## *BDNF* methylation and depression

**Fig. 1:**
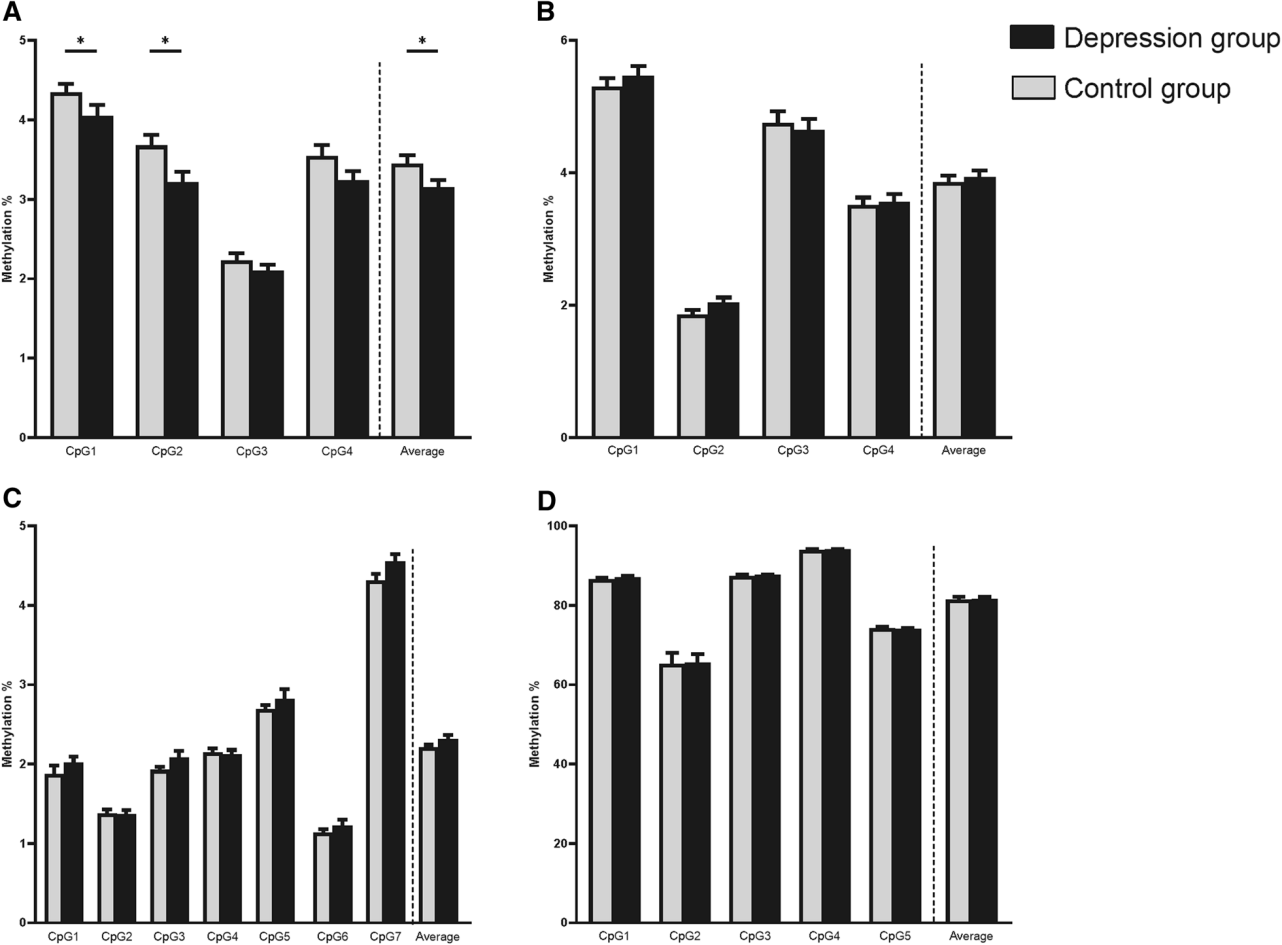
Overview of *BDNF* methylation in the control group (grey) and the depression group (black). DNA methylation is presented as mean and standard error of the mean for each of the analysed regions: promoter Ia (**A**), promoter Ib (**B**), promoter IV (**C**) and exon IX (**D**). Methylation of each individual CpG as well as the average methylation levels across all CpGs are presented for each analysed region. Significance levels are derived from Mann–Whitney *U* test (*p < 0.05, **p < 0.01)

When comparing the average methylation levels of different *BDNF* regions between the depression and the control group (Fig. 1; Additional file 1: Table S3), we observed significantly decreased methylation of *BDNF* exon Ia in the depression group (mean difference 0.30%, *p* = 0.042), as well as 2 individual CpGs (CpG1: mean difference 0.29%, *p* = 0.040, CpG2: mean difference 0.46%, *p* = 0.013). The group effect on average methylation of exon Ia remained significant when controlling for the Val66Met polymorphism (*B* = 0.282, *p* = 0.043, *η*_*p*_^2^ = 0.03).

As a confirmatory analysis, we tested whether the observed methylation differences were associated dimensionally with clinical features such as anhedonia and depression severity (Additional file 1: Table S4). In the overall sample, average methylation of *BDNF* exon Ia was negatively associated with anhedonia (Spearman's Rho = − 0.180, *p* = 0.036) and negative affect (Spearman's Rho = − 0.198, *p* = 0.021). In the depression group, no associations were observed (all *ps* > 0.05).

For exon Ib, IV and IX, there were no significant group differences (all *ps* > 0.05, results presented in Fig. 1 and Additional file 1: Table S3). In the dimensional analysis (Additional file 1: Table S4), average methylation of exon Ib was negatively associated with anhedonia in the overall sample (Spearman's Rho = − 0.177, *p* = 0.039) and in the MDD group separately (Spearman's Rho = − 0.225, *p* = 0.047) and was positively associated with positive affect in the depression group (Spearman's Rho = 0.232, *p* = 0.040). For exon IV, no associations were observed in the overall sample, but in the MDD group, average methylation was negatively associated with depression severity (Spearman's Rho = − 0.245, *p* = 0.031) and positively with positive affect (Spearman's Rho = 0.288, *p* = 0.011).

None of the average methylation levels differed between subjects with and without early life stress, neither in the overall sample nor among depressed patients (all *ps* > 0.05). Similarly, type of antidepressant did not have an impact on DNA methylation (all *ps* > 0.05).

## Val66Met, *BDNF* methylation and reward learning

In the overall sample, there was no significant effect of polymorphism on response bias (*F*(2,250) = 1.843, *p* = 0.136, *η*_*p*_^2^ = 0.015). However, DNA methylation of *BDNF* promoter Ib had a significant positive effect on reward bias (*F*(2,248) = 3.804, *p* = 0.026, *η*_*p*_^2^ = 0.030), indicating that participants with lower *BDNF* promoter Ib methylation showed lower response bias during the computerized reward learning task. More specifically, this association was driven by the effect of DNA methylation of this region on response bias in Block 1 (*B* = 0.040, *p* = 0.043) and 2 (*B* = 0.071, *p* = 0.008), but not Block 3 (*B* = − 0.004, *p* = 0.882). In addition, we did not observe a significant interaction effect between polymorphism and *BDNF* promoter Ib methylation on response bias (*F*(2,244) = 0.131, *p* = 0.865, *η*_*p*_^2^ = 0.001). DNA methylation of other *BDNF* regions (promoter Ia, promoter IV and exon IX) did not have a significant effect on the performance on the reward learning task (all *ps* > 0.05, data not shown).

**Fig. 2:**
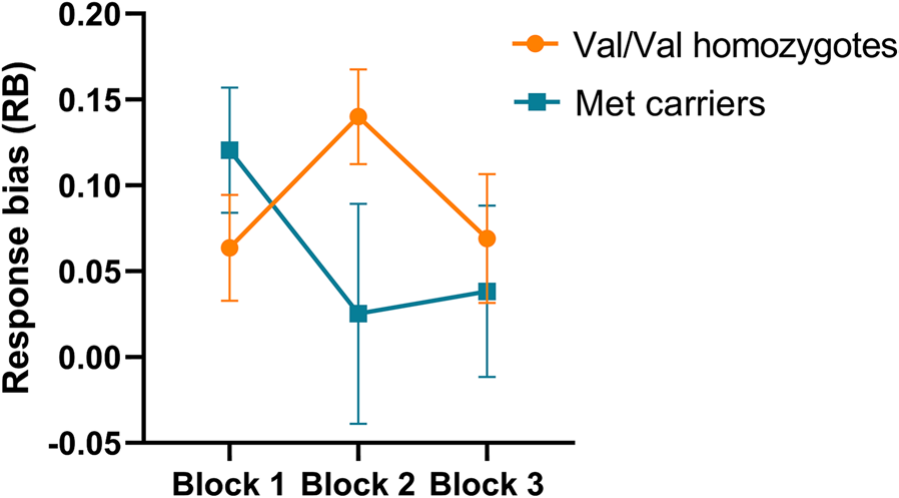
Comparison of reward bias as a function of blocks, as measured by the computerized reward learning task, between Val/Val homozygotes (orange) and Met carriers (blue) in the depression group. Data are presented as mean and standard error of the mean

In contrast, in the depression group, we observed a significant association between polymorphism and response bias (*F*(2,146) = 3.061, *p* < 0.050, *η*_*p*_^2^ = 0.040), whereby Met carriers had significantly lower response bias than Val/Val homozygotes, as illustrated in Fig. 2. Similar as in the overall sample, DNA methylation of *BDNF* promoter Ib had a significant positive effect on response bias (*F*(2,144) = 3.301, *p* = 0.040, *η*_*p*_^2^ = 0.044). More specifically, depressed patients with lower DNA methylation of promoter Ib showed lower response bias in Block 2 (*B* = 0.077, *p* = 0.015) but not in Block 1 and 3 (all *ps* > 0.05). When both predictors (polymorphism and promoter Ib methylation) were added to the model, only the effect of DNA methylation remained significant (*p* = 0.047) whereas the effect of polymorphism was lost (*p* = 0.072), indicating a potential indirect (mediating) effect of polymorphism on reward bias, via DNA methylation. When testing the interaction effect between polymorphism and DNA methylation on the performance on the reward learning task, no significant effect was found (*F*(2,140) = 1.635, *p* = 0.199, *η*_*p*_^2^ = 0.023). Type of antidepressant did not have a moderation effect on any of the observed associations (all *ps* > 0.05). DNA methylation of other BDNF regions did not have a significant effect on reward bias in the depression group (all *ps* > 0.05). The effect of polymorphism and *BDNF* promoter Ib methylation on response bias is presented in Table 2.

**Table 2:** Overview of the effect of polymorphism and *BDNF* promoter Ib methylation on reward bias analysed using repeated measures ANOVA

	Both groups			Depressed group			Control group		
	*F*	*p*	*η*_*p*_^2^	*F*	*p*	*η*_*p*_^2^	*F*	*p*	*η*_*p*_^2^
*Univariate models*									
Block*SNP	1.843	0.163	0.015	**3.061**	** < 0.05**	**0.040**	0.089	0.915	0.002
Block*Promoter Ib DNAm	**3.804**	**0.026**	**0.030**	**3.301**	**0.040**	**0.044**	0.471	0.626	0.009
*Multivariate model*									
Block*SNP	1.592	0.207	0.013	2.675	0.072	0.036	0.132	0.876	0.003
Block*Promoter Ib DNAm	**3.656**	**0.030**	**0.029**	**3.126**	**0.047**	**0.042**	0.508	0.603	0.010
*Interaction*									
Block*SNP	0.413	0.650	0.003	2.641	0.075	0.036	1.668	0.194	0.034
Block*Promoter Ib DNAm	**3.529**	**0.034**	**0.028**	**3.101**	**0.048**	**0.042**	0.525	0.593	0.011
Block*SNP*Promoter Ib DNAm	0.131	0.865	0.001	1.635	0.199	0.023	1.608	0.206	0.032

Significant predictors are marked in bold

## Val66Met, *BDNF* methylation and cognitive performance

**Fig. 3:**
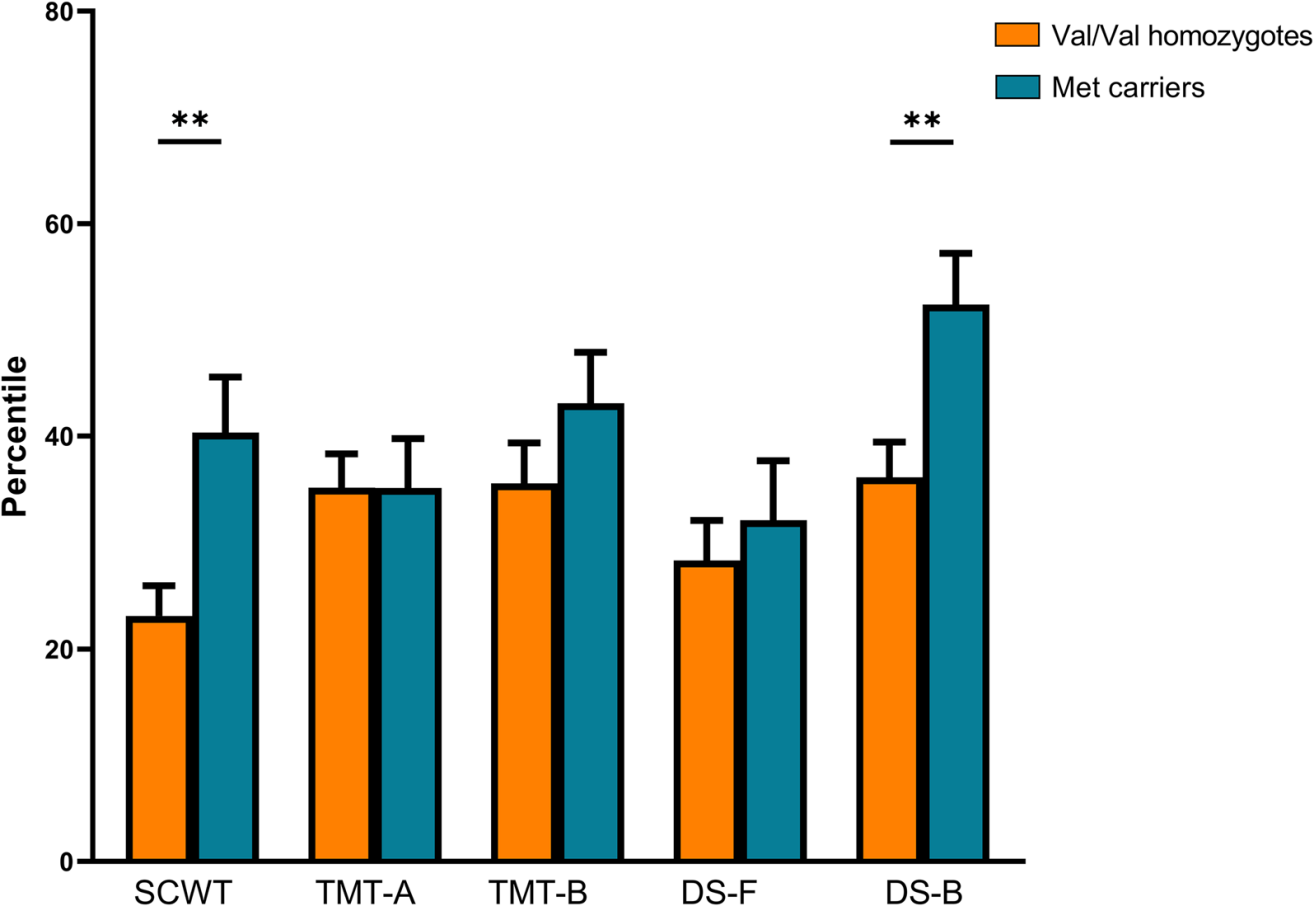
Comparison of cognitive performance between Val/Val homozygotes (orange) and Met carriers (blue) in the depression group. Data are presented as mean and standard error of the mean. Significance levels are derived from Mann–Whitney *U* test (*p < 0.05, **p < 0.01). SCWT: Stroop Color and Word Test; TMT-A: Trial Making Test part A; TMT-B: Trial Making Test part B; DS-F: Digit Span Forward; DS-B: Digit Span Backward

Regarding the effect of Val66Met on cognitive performance, there were no significant associations in the overall sample (all *ps* > 0.05). However, in the depression group, Met carriers had better performance on the SCWT (*Z* = 2.71, *p* = 0.007) and DS-B (*Z* = 2.62, *p* = 0.009), as presented in Fig. 3.

**Fig. 4:**
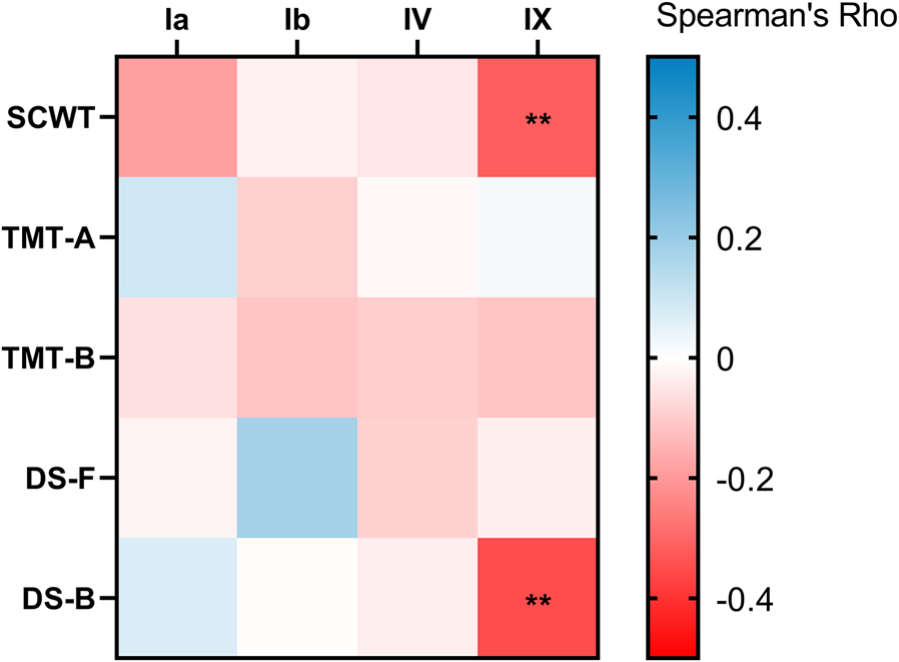
Correlation matrix (Spearman correlation) between *BDNF* methylation of different regions and cognitive performance in the depression group. Positive correlations are presented in blue whereas negative correlations are given in red and the intensity of colours corresponds to the values of Spearman’s Rho correlation coefficient. Significance: *p < 0.05, **p < 0.001. SCWT: Stroop Color and Word Test; TMT-A: Trial Making Test part A; TMT-B: Trial Making Test part B; DS-F: Digit Span Forward; DS-B: Digit Span Backward

In the overall sample, average methylation of different assays was not significantly associated with cognitive performance (all *ps* > 0.05). However, in depressed patients, average methylation of exon IX (containing the polymorphism) was negatively associated with performance of the SCWT (Spearman's Rho = − 0.228, *p* = 0.046) and DS-B (Spearman's Rho = − 0.334, *p* = 0.002), as illustrated in Fig. 4.

In addition, mediation analysis revealed that exon IX methylation was a significant mediator of the effect of Val66Met on DS-B (Effect 20.05, CI [1.47, 50.20]) but not SCWT (Effect 0.83 CI [− 29.88, 22.17]). The mediation effect of exon IX methylation on the association between Val66Met and DS-B is presented in Additional file 1: Fig. S5. Type of antidepressant did not moderate any of the observed associations in the depression group (all *ps* > 0.05).

## Discussion

In the present study, we probed the role of genetic and epigenetic regulation of *BDNF* in major depression, by focusing on anhedonia, reward learning and cognitive performance as the main outcomes. Our results demonstrated that lower *BDNF* methylation of promoter I contributed to more pronounced anhedonia and impaired reward learning in depressed patients. The presence of Met allele in the common *BDNF* polymorphism (Val66Met) had a negative effect on reward learning, but this seemed to be an indirect effect, via DNA methylation of promoter I. On the other hand, Met allele exhibited a protective function for cognitive performance (executive functioning in particular), through the mediating effect of reduced methylation in the *BDNF* coding region (exon IX) in the same population of depressed patients.

To the best of our knowledge, our study was the first to test the association between genetic and epigenetic control of *BDNF* in reward learning in MDD. Even though there are no other similar studies to compare these findings, the observed impairing effect of decreased *BDNF* methylation seems to be in line with the growing data demonstrating the depressogenic and anhedonic effects of BDNF in the mesolimbic reward circuitries in animals exposed to chronic social defeat stress [14, 15, 47]. In addition, the fact that we found a negative association between self-reported anhedonia and promoter I methylation as well as decreased methylation of this region in between-group comparison with a healthy population makes our conclusions stronger and more convincing. Interestingly, increased BDNF plasma levels have been previously associated with decreased reward learning in patients with bulimia nervosa [48]. Even though this is a different clinical and nosological phenotype from MDD, these data together with our findings provide evidence for the role of BDNF in reward circuitries disruption that extend beyond diagnostic categories. In the present study, we did not assess mRNA expression or serum/plasma BDNF levels and therefore our conclusions on the functional consequences of decreased *BDNF* promoter I methylation are limited. However, our previous work [46] together with other research [49] has shown a negative correlation between promoter I methylation and BDNF expression. In addition, we also observed association between depression severity and reduced positive affect with decreased promoter IV methylation, which was previously shown to correlate negatively with BDNF expression (16). This might be an additional indicator of increased BDNF expression in our population of depressed patients, even though direct association with anhedonia or reward learning was not found.

In our study, we also find a significant effect of genotype (Val66Met) on reward learning, whereby Met carriers showed a more blunted reward bias in the reward learning task than Val/Val homozygotes. We speculate that our findings are consistent with research indicating that the Met allele is a vulnerability allele for depression (and mental disease in general) due to its impairing effect on BDNF translocation and secretion [18]. However, as mentioned before, this conclusion failed to be replicated [17, 50, 51], perhaps indicating that this polymorphism determines vulnerability to environment-induced epigenetic changes rather than being a risk factor per se. Indeed, in our study, Val66Met was not associated with any of the outcomes in healthy participants, but only in the MDD group. In addition, this effect was lost after we controlled for DNA methylation of *BDNF* promoter I, indicating the predominant effect of epigenetic regulation and a potentially indirect (mediating) effect of the polymorphism. The importance of environment-induced epigenetic regulation of *BDNF* is also supported by evidence showing that BDNF signalling pathway does not play a role in reward circuitries in normal conditions, but gains importance once these are disrupted [52].

In contrast, among the MDD group, we found increased *BDNF* exon IX methylation to be associated with poorer executive functioning, particularly in the executive domain (STROOP and DS-B). To the best of our knowledge, methylation of this region has never been not tested before with respect to cognitive performance in depressed patients. Previously, Ferrer et al. [29] found methylation of exon I and IV to be associated with visual learning and memory, but reported no association with cognitive function tested, which is in line with our findings. Exon IX has been previously shown to be particularly abundant in the hippocampus [45] and de-methylation of this region was shown to induce adult hippocampal neurogenesis [53]. In addition, in our study, exon IX methylation was a mediator of the effect of Val66Met on the executive functions, whereby the presence of the Met allele appeared to be protective, which is somewhat surprising. Specifically, previous meta-analysis [54] have shown that Met carriers had lower hippocampal volume, but also provided evidence for publication bias and small effect sizes, questioning whether the observed effect is subject to a *winner's curse*. Moreover, some recent studies indicate that the Met allele can be protective for executive functions in the presence of depressive symptoms [55] and can promote recovery of executive functioning after PTSD [56], indicating a potentially differential effect of Val66Met on cognitive function in healthy individuals and in the presence of psychopathology. In our study, the effect of Val66Met on executive function was predominantly indirect, through DNA methylation of *BDNF* exon IX. Therefore, our results indicate that Val66Met is a vulnerability factor susceptible to epigenetic regulation and future studies should account for DNA methylation when further exploring the complex role of this polymorphism in different neural processes and psychopathology.

Several limitations need to be taken into account when interpreting the results of our study. First, almost all depressed patients were undergoing antidepressant treatment at the time of inclusion, which might have influenced *BDNF* methylation levels [25, 57]. Due to high collinearity with the MDD diagnosis, we did not include medication as a covariate in the analysis; however, we did test the effect of different types of medication on *BDNF* methylation levels and found no significant differences. Medication could have influenced the between-group differences in *BDNF* methylation, but it is highly unlikely that antidepressants could explain the observed correlations with anhedonia, reward learning and cognitive performance found in the MDD group. In addition, the effect of antidepressants on *BDNF* methylation remains unclear. Whereas studies show that antidepressants can restore reduced serum BDNF protein levels in depressed patients [8, 58], methylation studies show conflicting findings, demonstrating that antidepressants were associated with both increase and decrease in *BDNF* methylation in this patient population [25, 59, 60]. Moreover, some studies reported the absence of difference between *BDNF* methylation at the baseline and at the end of antidepressant treatment in depressed patients [61]. Therefore, we believe that methylation studies on both medication-free MDD patients as well as patients taking antidepressants are important complementary source of data as they allow comparing which epigenetic effects are potentially a result of medication intake and which reflect phenotypic changes linked to MDD itself. In the context of *BDNF*, this is still to be elucidated as we do not completely understand the effect of medication (and psychotherapy) on *BDNF* methylation patterns. Next, in the present study, we did not collect RNA samples and therefore mRNA expression analysis could not be performed. This is another limitation of the study as these data would help get a clearer picture of the direct effects of the observed DNA methylation changes on the gene expression and the overall interaction between genetic, epigenetic regulation and gene expression of *BDNF*. Previous data showed that both Val66Met and *BDNF* methylation of different exons could have an impact on *BDNF* expression [28, 30, 46] but further conclusions on this could not be drawn in our patient population. Next, in our study, we analysed DNA methylation using targeted approach (pyrosequencing), which allowed mapping DNA methylation patterns only in one part of the *BDNF* gene (20 individual CpGs). Other studies show Val66Met to have an influence on DNA methylation of other CpG position in the *BDNF* gene [27], and therefore, we might have failed to capture other genotype-DNA methylation interaction effects of relevance of the outcomes analysed here (reward learning and cognitive performance) in the population of clinically depressed patients. Finally, all patients included in the MDD group were hospitalized and therefore might be more prone to the chronic course of illness, which might limit generalizability of our findings.

## Conclusions

To conclude, our data suggest that Val66Met is associated with DNA methylation of different *BDNF* exons, which may in turn contribute to impaired reward learning and executive functioning in patients with MDD. We hypothesize that these genetic and epigenetic patterns affect reward learning and executive functioning in depressed patients via changes in the expression of different *BDNF* exons; however, this was not directly assessed in the present study and needs to be further explored. Therefore, we recommend future studies to simultaneously assess Val66Met, DNA methylation and possibly mRNA expression of different *BDNF* transcripts in order to learn more about their role in different pathways of relevance for MDD.

## Supplementary Information

**Additional file 1.** Overview of the pyrosequencing protocol, effect of genotype on DNA methylation and associations between DNA methylation and clinical variables.

## Data Availability

The datasets used and/or analysed during the current study are available from the corresponding author on reasonable request.

## References

[1] GBD (2017). Global, regional, and national incidence, prevalence, and years lived with disability for 354 diseases and injuries for 195 countries and territories, 1990–2017: a systematic analysis for the Global Burden of Disease Study 2017. Lancet.

[2] Gaynes B, Warden D, Trivedi M, Wisniewski S, Fava M, Rush J (2009). What did STAR∗D teach us? Results from a large-scale, practical, clinical trial for patients with depression. Psychiatry Serv.

[3] Menke A, Binder EB (2014). Epigenetic alterations in depression and antidepressant treatment. Dialogues Clin Neurosci.

[4] Duman RS, Monteggia LM (2006). A neurotrophic model for stress-related mood disorders. Biol Psychiatry.

[5] Binder D, Scharfman H (2004). Brain-derived neurotrophic factor. Growth Factors.

[6] Cunha C, Brambilla R, Thomas KL (2010). A simple role for BDNF in learning and memory?. Front Mol Neurosci.

[7] Molendijk ML, Spinhoven P, Polak M, Bus BAA, Penninx B, Elzinga BM (2013). Serum BDNF concentrations as peripheral manifestations of depression: evidence from a systematic review and meta-analyses on 179 associations (*N* = 9484). Mol Psychiatry.

[8] Sen S, Duman R, Sanacora G (2009). Serum BDNF, depression and anti-depressant medications: meta-analyses and implications. Biol Psychiatry.

[9] Fernandes B, Berk M, Turck C, Steiner J, Gonçalves C-A (2014). Decreased peripheral brain-derived neurotrophic factor levels are a biomarker of disease activity in major psychiatric disorders: a comparative meta-analysis. Mol Psychiatry.

[10] Lin P-Y (2009). State-dependent decrease in levels of brain-derived neurotrophic factor in bipolar disorder: a meta-analytic study. Neurosci Lett.

[11] Price RB, Duman R (2020). Neuroplasticity in cognitive and psychological mechanisms of depression: an integrative model. Mol Psychiatry.

[12] Koo JW, Chaudhury D, Han M, Nestler EJ (2019). Role of mesolimbic brain-derived neurotrophic factor in depression. Biol Psychiatry.

[13] Groves J (2007). Is it time to reassess the BDNF hypothesis of depression?. Mol Psychiatry.

[14] Berton O, Mcclung CA, Dileone RJ, Krishnan V, Renthal W, Russo SJ (2006). Essential role of BDNF in the mesolimbic dopamine pathway in social defeat stress. Science (80-.).

[15] Nestler EJ, Carlezon WA (2005). The mesolimbic dopamine reward circuit in depression. Biol Psychiatry.

[16] Pruunsild P, Sepp M, Orav E, Koppel I, Timmusk T (2011). Identification of cis-elements and transcription factors regulating neuronal activity-dependent transcription of human BDNF gene. J Neurosci.

[17] Tsai S-J (2018). Critical issues in BDNF Val66Met genetic studies of neuropsychiatric disorders. Front Mol Neurosci.

[18] Egan MF, Kojima M, Callicott JH, Goldberg TE, Kolachana BS, Bertolino A (2003). The BDNF val66met polymorphism affects activity-dependent secretion of BDNF and human memory and hippocampal function. Cell.

[19] Klengel T, Binder EB (2015). Review epigenetics of stress-related psychiatric disorders and gene 3 environment interactions. Neuron.

[20] Newell-price J, Clark AJL, King P (2000). DNA methylation and silencing of gene expression. Trends Endocrinol Metab.

[21] Bakusic J, Schaufeli W, Claes S, Godderis L (2017). Stress, burnout and depression: a systematic review on DNA methylation mechanisms. J Psychosom Res.

[22] Zheleznyakova GY, Cao H, Schiöth HB (2016). BDNF DNA methylation changes as a biomarker of psychiatric disorders: literature review and open access database analysis. Behav Brain Funct.

[23] Li M, D’Arcy C, Li X, Zhang T, Joober R, Meng X (2019). What do DNA methylation studies tell us about depression? A systematic review. Transl Psychiatry.

[24] Fuchikami M, Morinobu S, Segawa M, Okamoto Y, Yamawaki S, Ozaki N (2011). DNA methylation profiles of the brain-derived neurotrophic factor (BDNF) gene as a potent diagnostic biomarker in major depression. PLoS ONE.

[25] Carlberg L, Scheibelreiter J, Hassler MR, Schloegelhofer M, Schmoeger M, Ludwig B (2014). Brain-derived neurotrophic factor (BDNF)—epigenetic regulation in unipolar and bipolar affective disorder. J Affect Disord.

[26] Dell’Osso B, D’Addario C, Palazzo MC, Benatti B, Camuri G, Galimberti D (2014). Epigenetic modulation of BDNF gene: differences in DNA methylation between unipolar and bipolar patients. J Affect Disord.

[27] Gaunt TR, Shihab HA, Hemani G, Min JL, Woodward G, Lyttleton O (2016). Systematic identification of genetic influences on methylation across the human life course. Genome Biol.

[28] Sheikh HI, Hayden EP, Kryski KR, Smith HJ, Singh SM (2010). Genotyping the BDNF rs6265 (val66met) polymorphism by one-step amplified refractory mutation system PCR. Psychiatr Genet.

[29] Ferrer A, Labad J, Salvat-Pujol N, Barrachina M, Costas J, Urretavizcaya M (2019). BDNF genetic variants and methylation: effects on cognition in major depressive disorder. Transl Psychiatry.

[30] Polli A, Ghosh M, Bakusic J, Ickmans K, Monteyne D, Velkeniers B (2020). DNA methylation and BDNF expression account for symptoms and widespread hyperalgesia in patients with Chronic Fatigue Syndrome and Fibromyalgia. Arthritis Rheumatol.

[31] Hsieh M, Lin C, Lee C, Huang T (2019). Abnormal brain-derived neurotrophic factor Exon IX promoter methylation, protein, and mRNA levels in patients with major depressive disorder. J Clin Med.

[32] Vrieze E, Demyttenaere K, Bruffaerts R, Dirk H, Pizzagalli D, Sienaert P (2014). Dimensions in major depressive disorder and their relevance for treatment outcome. J Affect Disord.

[33] Vrieze E, Pizzagalli D, Demyttenaere K, Hompes T, Sienaert P, de Boer P (2013). Reduced reward learning predicts outcome in major depressive disorder. Biol Psychiatry.

[34] First MB, Spitzer RL, Gibbon M, Williams JBW. Structured clinical interview for DSM-IV-TR axis I disorders, research version, non-patient edition, for DSMIV. 2002.

[35] Hamilton M (1960). A rating scale for depression. J Neurol Neurosurg Psychiatry.

[36] Draijer N, Langeland W (1999). Childhood trauma and perceived parental dysfunction in the etiology of dissociative symptoms in psychiatric inpatients. Am J Psychiatry.

[37] Snaith RP, Hamilton M, Morley S, Humayan A, Hargreaves D, Trigwell P (1995). A scale for the assessment of hedonic tone the Snaith–Hamilton Pleasure Scale. Br J Psychiatry.

[38] Watson D, Clark LA, Tellegen A (1988). Development and validation of brief measures of positive and negative affect: the PANAS scales. J Pers Soc Psychol.

[39] Stroop JR (1992). Studies of interference in serial verbal reactions. J Exp Psychol Gen.

[40] Tombaugh TN (2004). Trail making test A and B: normative data stratified by age and education. Arch Clin Neuropsychol.

[41] Wechsler D (2008). Wechsler Adult Intelligence Scale-Fourth Edition: administration and scoring manual.

[42] Van Der Elst W, Van Boxtel MPJ, Van Breukelen GJP, Jolles J (2006). The stroop color-word test: Influence of age, sex, and education; and normative data for a large sample across the adult age range. Assessment.

[43] Pizzagalli D, Iosifescu D, Hallett LA, Ratner KG, Fava M (2009). Reduced hedonic capacity in major depressive disorder: evidence from a probabilistic reward task. J Psychiatr Res.

[44] Pizzagalli DA, Jahn AL, O’Shea JP (2008). Toward an objective characterization of an anhedonic phenotype: a signal-detection approach. Biol Psychiatry.

[45] Cattaneo A, Cattane N, Begni V, Pariante CM, Riva MA (2016). The human BDNF gene: peripheral gene expression and protein levels as biomarkers for psychiatric disorders. Transl Psychiatry.

[46] Bakusic J, Ghosh M, Polli A, Schaufeli W, Claes S, Godderis L (2020). Epigenetic perspective on the role of brain-derived neurotrophic factor in burnout. Transl Psychiatry.

[47] Eisch AJ, Bolan CA, De WJ, Simonak RD, Pudiak CM, Barrot M (2003). Brain-derived neurotrophic factor in the ventral midbrain—nucleus accumbens pathway: a role in depression. Biol Psychiatry.

[48] Homan P, Grob S, Milos G, Schnyder U, Eckert A, Lang U (2015). The role of BDNF, leptin, and catecholamines in reward learning in bulimia nervosa. Int J Neuropsychopharmacol.

[49] D’Addario C, Dell’Osso B, Galimberti D, Palazzo MC, Benatti B, Di Francesco A (2013). Epigenetic modulation of BDNF gene in patients with major depressive disorder. Biol Psychiatry.

[50] Verhagen M, van der Meij A, van Deurzen P, Janzing J, Arias-Vásquez A, Buitelaar J (2010). Meta-analysis of the BDNF Val66Met polymorphism in major depressive disorder: effects of gender and ethnicity. Mol Psychiatry.

[51] Notaras M, Hill R, Van Den Buuse M (2015). The BDNF gene Val66Met polymorphism as a modifier of psychiatric disorder susceptibility: progress and controversy. Mol Psychiatry.

[52] Russo SJ, Nestler EJ (2013). The brain reward circuitry in mood disorders. Nat Rev Neurosci.

[53] Ma DK, Jang M, Guo JU, Kitabatake Y, Pow-anpongkul N, Flavell RA (2009). Neuronal activity-induced Gadd45b promotes epigenetic DNA demethylation and adult neurogenesis. Science (80-.).

[54] Molendijk ML, Bus BAA, Spinhoven P, Kaimatzoglou A, Voshaar RCO, Penninx BWJH (2012). A systematic review and meta-analysis on the association between BDNF val 66 met and hippocampal volume—a genuine effect or a winners curse?. Am J Med Genet Part B Neuropsychiatr Genet.

[55] Sanwald S, Montag C, Kiefer M (2020). depressive emotionality moderates the influence of the BDNF Val66Met polymorphism on executive functions and on unconscious semantic priming. J Mol Neurosci.

[56] Krueger F, Pardini M, Huey ED, Raymont V, Solomon J, Lipsky RH (2011). The role of the Met66 brain-derived neurotrophic factor allele in the recovery of executive functioning after combat-related traumatic brain injury. J Neurosci.

[57] Lee B, Kim Y (2010). The roles of BDNF in the pathophysiology of major depression and in antidepressant treatment. Psychiatry Investig.

[58] Brunoni R, Lopes M, Fregni F (2008). A systematic review and meta-analysis of clinical studies on major depression and BDNF levels: implications for the role of neuroplasticity in depression. Int J Neuropsychopharmacol.

[59] Wang P, Zhang C, Lv Q, Bao C, Sun H, Ma G (2018). Association of DNA methylation in BDNF with escitalopram treatment response in depressed Chinese Han patients. Eur J Clin Pharmacol.

[60] Webb LM, Phillips KE, Ho MC, Veldic M, Blacker CJ (2020). The relationship between DNA methylation and antidepressant medications: a systematic review. Int J Mol Sci.

[61] Tadić A, Müller-Engling L, Schlicht K, Kotsiari A, Dreimüller N, Kleimann A (2014). Methylation of the promoter of brain-derived neurotrophic factor exon IV and antidepressant response in major depression. Mol Psychiatry.

