# Supplementary material for: Interplay of Val66Met and BDNF methylation: effect on reward learning and cognitive performance in major depression
Source: Clin Epigenetics. 2021 Jul 29;13:149. doi: 10.1186/s13148-021-01136-z (PMC8323304; doi:10.1186/s13148-021-01136-z)
Supplement: Supplementary file 1 — Additional file 1. Overview of the pyrosequencing protocol, effect of genotype on DNA methylation and associations between DNA methylation and clinical variables. [file 13148_2021_1136_MOESM1_ESM.docx]

PCR and sequencing primers

To assess DNA methylation in promoter of exon I, we used commercially available assays designed by QIAGEN and we report the product codes and analysed sequences. For DNA methylation of promoter of exon IV and the coding region of exon IX, we designed PCR and sequencing primers using PyroMark Assay Design SW 2.0 (Qiagen).

PCR cycling conditions for all primers were as follows:

- Activation step: 95 °C for 15'
- 45 cycles including 3 steps
  - Denaturation: 94 °C for 30''
  - Annealing step with temperature indicated in Table S1. for 30''
  - Extension: 72 °C for 30''
- Final extension step: 72 °C for 10'

Assay validation, reproducibility analysis and the pyrosequencing protocol were described in detail previously^[[1]](#footnote-1)^.

**Table S1.** Overview of analysed sequences, genomic locations, PCR and sequencing primers for DNA methylation analysis

| **Region** | **Analysed sequence** | **Genomic location^a^** | **PCR primers** | **Sequencing primer** | **Number of CpGs** | **Genomic location of CpGs^a^** | **Annealing temperature** |
| --- | --- | --- | --- | --- | --- | --- | --- |
| Promoter of exon Ia | CTG CAT G**CG** T**CG** AAG **CGC G**A | 27743853-27743863 | P8_PM00155540 | P8_PM00155540 | 4 | CpG1: 27743853  CpG2: 27743856  CpG3: 27743861  CpG4: 27743863 | 56 °C |
| Promoter of exon Ib | TTA CTT TC**C** **G**CC AAC A**CG** TGA CCT CTT **CG**C TTC CCA GCT TG**C G**T | 27744313-27744346 | P9_PM00155547 | P9_PM00155547 | 4 | CpG1: 27744345  CpG2: 27744337  CpG3: 27744326  CpG4: 27744312 | 56 °C |
| Promoter of exon IV | TTA T**CG** **CG**G AGA GGG TTG TTT T**CG** TTG T**CG** TTT TTT T**CG** G**CG** AAT TAG TAT GAA ATT TTT TTG TTT TTG T**CG** AGA TTA AAT GGA GTT TTT | 27723076-27723165 | Forward: GGG TTG GAA GTG AAA ATA TTT GTA AA  Reverse: /5Biosg/CC CCA TCA ACC AAA AAC TCC ATT TAA TC | GTG GAT TTT TAT TTA TTT TTT TAT | 7 | CpG1: 27723161  CpG2: 27723159  CpG3: 27723143  CpG4: 27723137  CpG5: 27723128  CpG6: 27723125  CpG7: 27723095 | 54 °C |
| Coding region of exon IX | TAT TAT TGG TTG ATA TTT T**CG** AAT A**CG/A** TGA TAG AAG AGT TGT TGG ATG AGG ATT AGA AAG TT**C** **G**GT TTA ATG AAG AAA ATA ATA AGG A**CG** TAG ATT TGT ATA **CG**T TTA GGG TGA TGT TTA GTA GTT AA | 27679815- 27679941 | Forward: ATG AAG GTT GTT TTT ATG AAA GAA GTA  Reverse: /5Biosg/AC CCA CTC ACT AAT ACT A | AGG TTT AAG AGG TTT GA | 5 | CpG1: 27679923  CpG2: 27679917  CpG3: 27679880  CpG4: 27679854  CpG5: 27679840 | 54 °C |

^a^ As per hg19 nomenclature. Determined using iMETHYL database (<http://imethyl.iwate-megabank.org>).

**Table S2.** Differences in *BDNF* methylation between Val/Val homozygotes and Met carriers in the overall sample (N=138) tested using Mann-Whitney *U* test.

|  | Val/Val | Met carriers | Group differences | |
| --- | --- | --- | --- | --- |
|  | Mean rank | Mean rank | Z | *p*-value |
| Promoter exon Ia |  |  |  |  |
| **Average** | **75.36** | **55.93** | **-2.748** | **0.006** |
| **CpG1** | **75.51** | **55.65** | **-2.810** | **0.005** |
| **CpG2** | **74.10** | **58.23** | **-2.245** | **0.025** |
| CpG3 | 72.34 | 61.46 | -1.539 | 0.124 |
| **CpG4** | **74.66** | **57.21** | **-2.468** | **0.014** |
| Promoter exon Ib |  |  |  |  |
| **Average** | **74.53** | **57.09** | **-2.455** | **0.014** |
| CpG1 | 72.82 | 60.32 | -1.760 | 0.078 |
| CpG2 | 70.58 | 64.56 | -0.847 | 0.397 |
| **CpG3** | **74.21** | **57.69** | **-2.325** | **0.020** |
| **CpG4** | **73.56** | **58.93** | **-2.059** | **0.039** |
| Promoter exon IV |  |  |  |  |
| Average | 70.61 | 61.54 | -1.284 | 0.199 |
| CpG1 | 70.73 | 61.32 | -1.333 | 0.182 |
| CpG2 | 71.24 | 60.35 | -1.542 | 0.123 |
| CpG3 | 69.39 | 63.88 | -0.780 | 0.435 |
| CpG4 | 66.47 | 69.48 | -0.426 | 0.670 |
| **CpG5** | **73.64** | **55.75** | **-2.533** | **0.011** |
| CpG6 | 68.99 | 64.65 | -0.614 | 0.539 |
| CpG7 | 67.20 | 68.08 | -0.124 | 0.901 |
| Coding region of exon IX |  |  |  |  |
| **Average** | **93.43** | **24.63** | **-9.629** | **<0.001** |
| **CpG1** | **74.88** | **59.42** | **-2.164** | **0.030** |
| **CpG2** | **93.50** | **24.50** | **-9.656** | **<0.001** |
| **CpG3** | **79.21** | **51.30** | **-3.905** | **<0.001** |
| **CpG4** | **79.22** | **51.28** | **-3.909** | **<0.001** |
| CpG5 | 69.35 | 69.78 | -0.060 | 0.952 |

**Table S3.** Between group differences in *BDNF* methylation tested using Mann-Whitney *U* test.

|  | Control group | MDD group | Group differences | |
| --- | --- | --- | --- | --- |
|  | Mean rank | Mean rank | Z | *p*-value |
| Promoter exon Ia |  |  |  |  |
| **Average** | **76.47** | **62.57** | **-2.035** | **0.042** |
| **CpG1** | **76.54** | **62.52** | **-2.053** | **0.040** |
| **CpG2** | **78.28** | **61.22** | **-2.497** | **0.013** |
| CpG3 | 71.64 | 66.17 | -0.801 | 0.423 |
| **CpG4** | **74.72** | **63.88** | **-2.035** | **0.042** |
| Promoter exon Ib |  |  |  |  |
| Average | 68.87 | 68.23 | -0.093 | 0.926 |
| CpG1 | 65.44 | 70.71 | -0.770 | 0.442 |
| CpG2 | 61.42 | 73.61 | -1.780 | 0.075 |
| CpG3 | 72.33 | 65.73 | -0.964 | 0.335 |
| CpG4 | 68.82 | 68.27 | -0.082 | 0.935 |
| Promoter exon IV |  |  |  |  |
| Average | 64.23 | 69.85 | -0.826 | 0.409 |
| CpG1 | 60.55 | 72.49 | -1.755 | 0.079 |
| CpG2 | 69.41 | 66.13 | -0.483 | 0.629 |
| CpG3 | 63.97 | 70.03 | -0.891 | 0.373 |
| CpG4 | 70.32 | 65.47 | -0.713 | 0.476 |
| CpG5 | 68.69 | 68.06 | -0.300 | 0.764 |
| CpG6 | 66.72 | 68.06 | -0.196 | 0.844 |
| CpG7 | 60.27 | 72.69 | -1.827 | 0.068 |
| Coding region of exon IX |  |  |  |  |
| Average | 68.95 | 69.90 | -0.138 | 0.890 |
| CpG1 | 62.46 | 74.61 | -1.762 | 0.078 |
| CpG2 | 68.91 | 69.93 | -0.147 | 0.883 |
| CpG3 | 65.50 | 72.40 | -1.001 | 0.317 |
| CpG4 | 69.05 | 69.83 | -0.112 | 0.911 |
| CpG5 | 71.39 | 68.13 | -0.472 | 0.637 |

**Table S4.** Summary of Spearman correlations between depressive symptoms and *BDNF* methylation in both groups and in MDD patients

|  | Both groups (N=138) | | | | MDD group (N=80) | | | |
| --- | --- | --- | --- | --- | --- | --- | --- | --- |
|  | Depression severity (HDRS) | Anhedonia (SHAPS) | Positive affect (PANAS) | Negative affect (PANAS) | Depression severity (HDRS) | Anhedonia (SHAPS) | Positive affect (PANAS) | Negative affect (PANAS) |
| BDNF methylation |  |  |  |  |  |  |  |  |
| Promoter Ia | -0.160 | **-0.180*** | 0.117 | **-0.198*** | -0.061 | -0.003 | -0.026 | -0.043 |
| Promoter Ib | -0.059 | **-0.177*** | 0.103 | -0.021 | -0.150 | **-0.225*** | **0.232*** | 0.042 |
| Promoter IV | -0.022 | -0.015 | 0.057 | 0.006 | **-0.245*** | -0.078 | **0.288*** | 0.047 |
| Exon IX | -0.048 | 0.031 | 0.050 | -0.009 | -0.050 | 0.011 | 0.119 | -0.075 |

HDRS: Hamilton Rating Scale for Depression; PANAS: Positive and Negative Affect Scale; SHAPS: Snaith­Hamilton Pleasure Scale

**p*<0.05

**Figure S5.** Mediation analysis testing mediating effect of *BDNF* exon IX on the association between Val66Met polymorphism and cognitive performance (Digit Span Backward) in the depression group.

**Total effect** (c) of Val66Met on cognitive performance=16.22, SE=5.67, *t*=2.86, *p*=0.0054

**Direct effect** (c') of Val66Met on cognitive performance=-3.82, SE=13.60, *t*=-0.28, *p*=0.7794

**Indirect effect** (ab) of Val66Met on cognitive performance=20.05, Bootstrapped SE=12.0, Lower Bootstrapped CI=0.94; Upper Bootstrapped CI=49.37

DNAm: DNA methylation; DS-B: Digit Span Backward

**
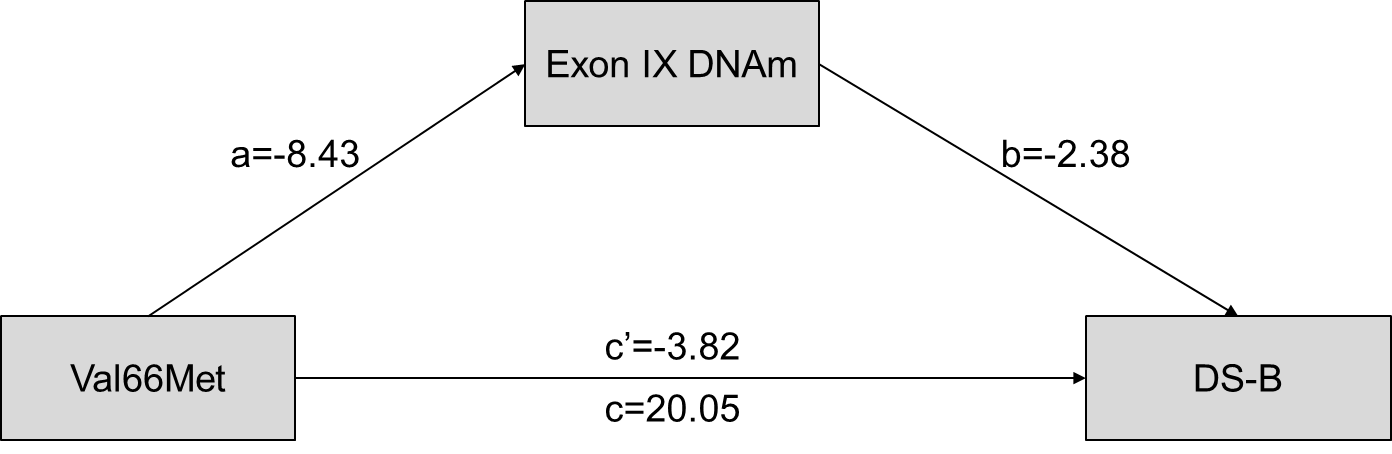
**

1. Bakusic J, Ghosh M, Polli A, Schaufeli W, Claes S, Godderis L. Epigenetic perspective on the role of brain-derived neurotrophic factor in burnout. Transl Psychiatry. 2020; 10:354. [↑](#footnote-ref-1)
